# Periodontology: The Past, the Present, the Future

**DOI:** 10.1111/jre.13370

**Published:** 2024-12-11

**Authors:** Jan Lindhe, Mario Romandini

**Affiliations:** ^1^ Department of Periodontology Sahlgrenska Academy at University of Gothenburg Gothenburg Sweden; ^2^ Department of Periodontology, Faculty of Dentistry University of Oslo Oslo Norway

**Keywords:** biofilms, dental implants, diagnosis, forecasting, guided tissue regeneration, history, periodontal diseases

In 2025, the *Journal of Periodontal Research* proudly celebrates its 60th anniversary. On this occasion, after more than 50 years, we are transitioning to 12 issues per year [1]. Together with this milestone, we introduce one of the exciting initiatives marking the journal's new era: a series of Perspective Articles under the theme “The Past, The Present, The Future”. This series will feature contributions from the “giants” of our field and will serve as a unique opening to each issue of the *Journal of Periodontal Research*.

The series commences with an insightful contribution from one of the most influential figures ever in periodontology: Professor Jan Lindhe. This manuscript stems from a memorable conversation we shared over tea and biscuits at his home in May 2024. During this magnetic interview, I was transported back in time to relive the pivotal moments that have shaped periodontology, vividly recounted from his extraordinary memory. At the same time, I was struck by his revolutionary mindset, as he continues to challenge established dogmas and share his visionary ideas for the future of our discipline.

I am delighted to share the essence of this unforgettable encounter, which holds a special place in my heart. I hope that this manuscript not only transports readers to that tea‐and‐biscuits moment, but also ignites a burst of inspiration from the past, present, and future of periodontology.

Mario Romandini

Editor‐in‐Chief

*Journal of Periodontal Research*

## 1 The Past

The launch of the *Journal of Periodontal Research* in the mid‐1960s marked a transformative moment for periodontology. At the time, Dr. Harald Löe—an influential periodontist and researcher—recognized the need for a dedicated journal that would treat periodontology as a research‐driven discipline. Unlike the clinical‐focused publications of the time, which centered on practical tips and specific geographic areas, this new journal aimed to elevate scientific inquiry in periodontology, positioning the field as a serious, research‐based specialty globally. For many years, the *Journal of Periodontal Research* earned its place as one of the leading journals in dentistry worldwide.

My own career began alongside the *Journal of Periodontal Research*. My first article as lead author was published in JPR in 1966 [2], the second ever piece published in the journal. Periodontology was still finding its footing then, and at that time, the main focus was on understanding the etiology of periodontitis.

### 1.1 Phase 1: The Role of Plaque in Periodontitis

I had just begun working in Sweden at Malmö University, where Dr. Hilding Björn, head of oral surgery, had recently turned his interest toward periodontology. Dr. Björn's attention had been drawn to the work coming out of Oslo from Dr. Jens Waerhaug, whose research had, for the first time, brought periodontal pathology firmly into the clinical spotlight.

Waerhaug's 1952 thesis, *The Gingival Pocket* [3], was groundbreaking. His observations on the intimate relationship between plaque, calculus, and the inflammatory lesions surrounding them were precise and, for that time, radical. Waerhaug went even further by demonstrating that periodontitis was not primarily a bone disease, but a pathology affecting the soft tissue and connective structures surrounding the teeth. The disease is in the connective tissue, and the bone just moves away.

After that, Waerhaug made the logical next step forward [4]. He examined around 1500 factory workers across different age groups in Oslo, meticulously recording their oral hygiene and periodontal health. Over five years, Waerhaug and his team systematically removed plaque and calculus, offering consistent oral hygiene instructions to each subject. The results were definitive—routine plaque removal dramatically reduced both gingivitis and tooth loss. This work laid the foundation for what I consider “Phase 1” in periodontology: the understanding of periodontitis, where plaque and calculus were recognized as primary etiological agents.

Soon after, Dr. Löe himself made his landmark contribution with his 1965–66 series: *Experimental Gingivitis in Man* [5, 6, 7, 8] (three of them published in the *Journal of Periodontal Research*). We had a study group between Malmö and Aarhus that met occasionally to exchange views. In one of these meetings, Löe's group presented this experimental gingivitis study, which was a logical follow‐up to Waerhaug's findings. Their study showed a clear, direct relationship between plaque accumulation and the onset of gingivitis, as well as between plaque removal and the resolution of inflammation. I vividly recall sitting in the audience during Löe's presentation, just behind Dr. Björn. The atmosphere in the room was electric; there was a palpable sense that something fundamental had changed forever. We were no longer just speculating: we had hard data that established plaque as the root cause of gingival inflammation.

Around the same time, chlorhexidine emerged as a promising antiseptic agent. Dr. Löe introduced chlorhexidine at a research meeting in Rochester in 1970, demonstrating that gingivitis could be entirely prevented in experimental settings with its use [9]. For a moment, we believed chlorhexidine would revolutionize dental care. Then, Muehlemann and Schroeder from Zurich claimed that this was not a new discovery, as they had used the substance before, but had called it something different. However, in the end, the practical impact of chlorhexidine proved less far‐reaching. While chlorhexidine took a role, it didn't replace the need for mechanical plaque control and, ultimately, didn't transform the field as some of us had initially hoped.

This period also saw a contentious debate over whether occlusal trauma could contribute to periodontitis. The Scandinavian school, including myself, argued that plaque was the dominant factor, while Glickman and Smulow in the United States suggested that occlusal trauma played the most significant role. These competing views—the Scandinavian concept versus the Glickman concept—were named as such for many years. To me, their evidence wasn't strong. In their autopsy material, Glickman and Smulow described how mobile teeth had more angular defects compared to adjacent non‐mobile teeth, concluding that traumatic occlusion caused clinical attachment loss [10]. The same argument was put forward by Pihlstrom from Minnesota in their famous study on maxillary first molars [11]. Their argument was logical, but it relied on cross‐sectional data. If you read the data from left to right, yes, trauma seemed important. But if you read it from right to left—angular defects mean more attachment loss, which means more mobility. We then performed animal studies that showed how trauma alone, without plaque, rarely lead to significant periodontal damage [12, 13, 14]. We never claimed that occlusion wasn't important, but we firmly established plaque as the central etiological factor.

The next step was to establish a treatment protocol based on systematic plaque control, which required longitudinal studies. Now, I have to be a bit selfish in stating that this included the “Göteborg approach”: systematically treating large groups of patients, following up on different procedures, and evaluating their effectiveness. During the observation period, patients were placed under a strict plaque control regimen [15]. We demonstrated that the essential factor was not the specific technique used to eliminate the pocket or treat the root surface, but rather the elimination of plaque and calculus. If I may, I believe this was one of my most important contributions to periodontology.

### 1.2 Phase 2: Guided Tissue Regeneration—The Beginnings of Periodontal Regeneration

The next major breakthrough emerged in the 1970s with the concept of periodontal regeneration. I remember a turning point in 1976, during a visit to the Eastman Dental Center in Rochester, where I saw a study by Dr. Jack Caton and Dr. Helmut Zander [16]. They had induced periodontitis in monkeys using orthodontic elastics and then surgically instrumented the roots. The radiographs showed substantial bone regrowth. However, in the tissue sections, a junctional epithelium was seen facing both the root surface and the bone. This finding challenged everything we had previously learned. Up to that point, we believed that bone regrowth was synonymous with periodontal regeneration. We had even published numerous papers equating bone fill with regeneration [17, 18], but Caton and Zander's study suggested this evidence was insufficient: they demonstrated that epithelium could exist between the bone and the root surface. In parallel, Gary Armitage demonstrated that when probing an inflamed pocket, the probe could penetrate beyond the inflammatory infiltrate, whereas in a healthy pocket, it would not reach the bottom of the epithelium [19]. Together, these findings called into question everything we thought we knew about new attachment.

Around this time, two brilliant minds, Sture Nyman and Thorkild Karring arrived in Gothenburg. Nyman had been there since 1969, and Karring joined in the mid‐1970s. They were both exceptionally intelligent, enjoyed working together, and focused on understanding which cells could produce cementum. A key line from a report by Tony Melcher in Canada became the focal point for our research in Gothenburg in the late 1970s: *the cells that populate the periodontal wound determine the quality of healing* [20]. Nyman and Karring started investigating whether different cell types—bone cells, gingival connective tissue cells, and periodontal ligament cells—could produce cementum. Together with some graduate students, they advanced the concept through extensive animal studies [21, 22, 23] and a case report [24]. The results were clear: bone and gingival cells could not produce cementum, but periodontal ligament cells could. This finding laid the groundwork for guided tissue regeneration. Years later, Maurizio Tonetti and his team demonstrated that while guided tissue regeneration works, the success varies significantly among clinicians [25, 26].

### 1.3 Phase 3: The Advent of Dental Implants

The arrival of dental implants marked yet another pivotal step in our field. I had the opportunity to work with Per‐Ingvar Brånemark long before implants were introduced. After completing my thesis, I developed an interest in pregnancy gingivitis—oddly enough, one always needs a research focus. This led me to investigate how estrogen and progesterone influence the inflammatory response. I conducted several studies with medical colleagues in the Department of Anatomy in Lund, who eventually suggested: “You should go to Dr. Brånemark in Gothenburg; he's an expert in microcirculation”. So, in 1964, I began collaborating with Brånemark, focusing on estrogen, progesterone, vascularization, and microvasculature. It was during this time that Brånemark began exploring dental implants, though I wasn't directly involved in that work. I also kept in touch with my former boss, Hilding Björn, whom I mentioned earlier. He was an oral surgeon and had removed many sub‐periosteal implants over his career, making him quite skeptical about implants. But eventually, he was proven wrong. Brånemark was exceptionally persistent, and despite numerous early failures—many of which we didn't know about at the time—the Brånemark method [27] ultimately gained global acceptance.

My involvement in implant dentistry began later. It was only in 1989, after returning from the United States, that we held a faculty meeting and decided it was time to investigate the potential and challenges of implants more thoroughly. In the USA, placing an implant in a fresh extraction socket was becoming popular, but we approached this trend more cautiously. We wondered: how could that approach work? Extracting a tooth causes dimensional changes in the alveolar ridge—would placing a metal implant in the fresh socket prevent these changes? This question led us, with our graduate students Mauricio Araujo and Giuseppe Cardaropoli, to study what happens after tooth extraction [28, 29, 30]. We observed significant changes, especially in buccal bone resorption, which prompted us to recommend caution with fresh socket implant placements. This research opened up new avenues in alveolar socket healing, socket grafting, and related studies—though that's another story.

This era also marked a shift as implant dentistry became integrated within periodontology, a field previously dominated by oral surgeons. We in Scandinavia helped drive the movement to incorporate implant treatments into periodontal practice, which transitioned the focus from mere implant survival to implant success, leading us to where we are today.

## 2 The Present

### 2.1 Challenge 1: Don't Forget Periodontitis!

Data indicate that oral health has improved up until around 2010, at least in Scandinavia [31]. With recent significant migration across Europe, there is uncertainty about how the dental health of newcomers might impact overall dental health [32]. Broadly speaking, there is anyway the perception that periodontal and caries conditions have gradually improved in developed countries, due to prevention efforts and regular dental visits. Perhaps for this reason, I sense a shift in periodontology today. I must be careful here, and I must emphasize that this is just a gut feeling. But it seems to me that periodontists are becoming less focused on preventing and treating periodontitis. Instead, they are increasingly drawn to advanced soft tissue grafting and periodontal or bone regeneration around teeth and implants. While these procedures undoubtedly benefit patients, they may be less critical for the long‐term longevity of the dentition. My concern is that, if this trend persists, periodontists may lose their skills in managing the core issue—periodontitis itself—potentially causing a setback in disease management.

### 2.2 Challenge 2: Peri‐Implantitis

Peri‐implantitis represents perhaps the most pressing clinical challenge in modern periodontology. In the early 1990s, Dr. Berglundh in Gothenburg and I conducted a preclinical study in dogs, where we observed that placing a ligature around both a tooth and an implant in the same animal produced notably different effects [33]. When I presented these findings at a meeting in southern Sweden, I cautioned that, while this phenomenon might not manifest in humans, if it did, we would face the question of how to treat it. We used the term “peri‐implantitis” at that time, but the reaction was dismissive: “this only happens in Gothenburg on the sixth floor”. We let it go, but soon reports of implant‐related problems began surfacing from others.

Dr. Berglundh and I attended an implant congress in Munich, where I presented our data on peri‐implantitis in dogs. By that time, we had conducted several studies on the topic. I addressed an audience of several hundred and asked: “Has anyone here seen this in patients?”. Silence. Many implant company representatives were in the room, so I asked to dim the lights and repeated the question. This time, the response was different: “yes, yes, yes”. People admitted seeing peri‐implantitis. It was clear that people were indeed aware this was happening but were hesitant to discuss it publicly. However, as with any hidden issue, it eventually came to light. Later, studies by Dr. Fransson and Dr. Derks in Gothenburg documented the issue [34, 35], proving that peri‐implantitis was real and required management.

Peri‐implantitis has a unique nature. While we can compare it to periodontitis, I believe there are additional factors at play that we still don't fully understand. Its destructive progression can be sudden and rapid. Standard plaque control protocols are not always effective; there is still an underlying factor that remains elusive.

## 3 The Future

### 3.1 Priority 1: Pathogenesis of Periodontitis

While placing metal in the jawbone can be beneficial for patients, it doesn't fully capture the essence of my curiosity (although maybe we shouldn't write that!). There's something intellectually fascinating—and still unresolved—within periodontology itself. Our understanding of the biofilm on the tooth surface has progressed; we can now identify which microorganisms inhabit deep pockets, but we still don't know whether these bacteria are responsible for pocket formation or if the pocket environment is simply conductive for that specific microbiota.

Moreover, our knowledge about the host response is frustratingly limited. Why do some people, with minimal visible gingival inflammation, experience severe attachment loss, while others with significant calculus and inflammation show little to no bone loss? Since the disease develops within the host, understanding the host response is essential, yet there's so much we still don't know. If I were younger, I would dedicate all my resources to unraveling the host's role—why some individuals react more intensely to the biofilm and where periodontitis begins. Moreover, why are certain teeth more vulnerable to periodontitis, even within the same mouth?

I don't believe periodontitis is a single disease. It may be more like diabetes, where similar outcomes occur but the underlying pathophysiology differs. We need to move beyond the old approach of focusing solely on plaque and periodontitis: those days are over. We need to be more sophisticated and include the host in our understanding, or we risk losing the game. Just as in diabetes, where some individuals with type 1 or type 2 diabetes face severe complications—even death—while others manage the disease with minimal issues. Within each type of diabetes, there are varied susceptibilities to complications, but the diseases are not identical. I believe the same holds true for periodontitis. The pathogenesis of periodontitis remains a fascinating, unfinished puzzle.

### 3.2 Priority 2: Diagnosis of Periodontitis

Years ago, I coined a term that I then sometimes regretted: I suggested distinguishing between periodontitis and “gingivitis with attachment loss”. This sparked controversy, yet my intent was to differentiate gingivitis with attachment loss, where the lesion is primarily in the gingiva, and attachment is slowly lost—little by little, but not at a level of significant concern. In contrast, periodontitis involves a more severe attachment loss, where teeth are lost. Perhaps it was a misstep, perhaps not.

In the past, we were concerned that dentists weren't using periodontal probes at all. Today, our worry has evolved: with a periodontal probe, we can't differentiate degrees of susceptibility. Currently, we can only identify susceptibility retrospectively, which is a major limitation. We need a predictive test that can reveal susceptibility early, not just in hindsight after bone loss has occurred. Consider a 28‐year‐old patient with over 50% bone loss—clearly susceptible to periodontitis. Now compare this with a 90‐year‐old patient showing similar bone loss. Both cases may share the same diagnosis, but they represent vastly different conditions.

In the past, we had to be dogmatic: “Control the plaque to control the disease”. And long‐term studies have largely validated this concept. However, these studies tended to include a diverse patient population as a single group and use mean values, ultimately masking individual susceptibilities. Under this generalized approach, critical distinctions between patients never surfaced. I believe the time has now come for a new generation to challenge all these dogmas and move periodontology forward.
